# Cardiovascular and electrocardiographic changes in Nigerians with a normal pregnancy

**DOI:** 10.5830/CVJA-2010-043

**Published:** 2011-04

**Authors:** PO Akinwusi, AA Akintunde, VO Oboro, AO Adeniji, IA Isawumi, RA Adebayo, MO Balogun, TO Ogungbamigbe

**Affiliations:** Department of Medicine, Ladoke Akintola University Teaching Hospital, Osogbo, Nigeria; Department of Medicine, Ladoke Akintola University Teaching Hospital, Osogbo, Nigeria; Department of Obstetrics and Gynaecology, Ladoke Akintola University Teaching Hospital, Osogbo, Nigeria; Department of Obstetrics and Gynaecology, Ladoke Akintola University Teaching Hospital, Osogbo, Nigeria; Department of Obstetrics and Gynaecology, Ladoke Akintola University Teaching Hospital, Osogbo, Nigeria; Department of Medicine, Obafemi Awolowo University Teaching Hospitals Complex, Ile-Ife, Nigeria; Department of Medicine, Obafemi Awolowo University Teaching Hospitals Complex, Ile-Ife, Nigeria; Department of Pharmacology and Therapeutics, Ladoke Akintola University Teaching Hospital, Osogbo, Nigeria

**Keywords:** cardiovascular, electrocardiographic changes, normal pregnancy, Nigeria

## Abstract

**Introduction:**

Pregnancy is associated with major haemodynamic and cardiac changes, which can mimic or precipitate cardiac diseases. There is a paucity of this kind of data among pregnant Nigerian women. This study was aimed at describing the cardiovascular and electrocardiographic changes found among healthy pregnant Nigerian women.

**Methods:**

This was an age-matched control study of 69 consecutive normal pregnant and 70 healthy non-pregnant controls. The study protocol included history, physical examination and 12-lead electrocardiography.

**Results:**

Diastolic blood pressure < 60 mmHg was significantly commoner among pregnant subjects than controls (64.7 vs 24.3%, respectively, *p* < 0.005). Mean heart rate was higher among pregnant women (88.34 ± 11.46 bpm) than the controls (75.16 ± 12.22 bpm, *p* = 0.020). Pregnant subjects also had a higher proportion of left ventricular hypertrophy (LVH) (10.2 vs 0%, *p* < 0.05) than non-pregnant controls. Abnormal cardiac findings included a loud second heart sound (P_2_), missed beats and systolic murmurs (41.2% in pregnant subjects vs 12.9% in non-pregnant controls, *p* < 0.05). Negroid-pattern ST-segment elevation was commoner among controls (24.3%) than pregnant subjects (2.9%, *p* < 0.005). Arrhythmias were rare among the study participants.

**Conclusion:**

Significant findings on examination were low diastolic blood pressure and a systolic ejection murmur. However, ECG changes showed a normal frontal-plane QRS axis, normal PR interval, significantly rare normal Negroidpattern ST elevation, significant LVH based on Araoye RI > 12 mm and a rarity of all forms of arrhythmias. These data may help resolve some cardiac diagnostic difficulties during pregnancy.

## Summary

Pregnancy is a normal physiological phenomenon causing major haemodynamic changes, including an increase in cardiac output, as well as sodium and water retention.^1^,^2^ In addition, these haemodynamic changes can mimic and/or precipitate cardiac diseases and cause diagnostic difficulties during pregnancy.

There is a paucity of data relating to cardiovascular (CV) and electrocardiographic (ECG) changes in healthy pregnant women in Nigeria, although the CV effects of pregnancy and associated ECG changes have been well documented outside Nigeria and Africa. Some of the notable ones are symptoms of exercise intolerance/left-sided heart failure, palpitations and syncope.^2^,^3^ Associated physical findings that have been reported include peripheral oedema and distended neck veins.^2^

Auscultatory examinations at different stages of pregnancy may reveal a loud first heart sound (S_1_) with exaggerated splitting, which may be mistaken for a fourth heart sound (S_4_) or systolic click;^2^ and a loud second heart sound (S_2_) with persistent splitting that may simulate a loud P_2_.^2^ The volume-overloaded state and the augmented blood flow may produce a physiological third heart sound (S_3_) and an ejection systolic murmur, respectively.^3^

ECG findings that have been reported include the QRS axis, which could be normal,^2^ right or left axis;^2^,^4^ frequent sinus tachycardia with accompanying shortened PR and QT intervals,^4^ as well as higher incidence of arrhythmias.^2^ Also reported are small Q waves and inverted P waves in lead III (abolished by inspiration), increased R/S ratio in leads V_1_ and V_2_, as well as sagging of ST segments and inversion of or flat T waves in lead III.^5^

This study was therefore undertaken to describe the CV and ECG changes in normal pregnancy among Nigerian women attending Ladoke Akintola University of Technology Teaching Hospital, Osogbo, south-west Nigeria.

## Methods

This was a cross-sectional, age-matched control study of 139 patients, comprising 69 consecutive healthy pregnant and 70 healthy non-pregnant patients at Ladoke Akintola University of Technology Teaching Hospital, Osogbo, Osun state, south-west, Nigeria. The study was carried out over a two-year period from February 2006 to January 2008. Institutional ethical clearance was obtained and all subjects gave informed consent.

All the patients were taken through a comprehensive study protocol of history and physical examination by two cardiologists. A 12-lead resting ECG with a long rhythm strip of lead II was recorded in all subjects. The same cardiologists independently reported each ECG.

Exclusion criteria included previous or current hypertension, diabetes mellitus, thyrotoxicosis, history suggestive of congenital or valvular heart disease, or any other form of cardiac disease, sickle cell disease and anaemia (PCV < 30%) at the time of recruitment.

Araoye^6^ and Sokolow-Lyon^7^ criteria were independently used to assess for left ventricular hypertrophy (LVH): Araoye criteria: R in lead I (RI) > 12 mm, or SV_2_ + RV_6_ ≥ 35 mm, with or without T-wave inversion/flattening in V_5_, V_6_. Sokolow-Lyon criteria: SV_1_ + RV_5_ (or RV_6_) ≥ 35 mm with or without T-wave inversion/flattening in V_5_, V_6_.

Corrected QT (QTc) was calculated for each patient using Bazett’s formula;^8^ a normal value in females is 0.37–0.44; > 0.44 is prolonged.^9^

In cases of incomplete data, an allowance was made for 10% fall-out from the analysis. Data were compared between the two groups. Data entry was into standard forms and statistical analysis was performed. All the tests were two-sided with a 0.05 significance level set. Differences in age, parity and estimated gestational age (EGA) were by *t*-test, with chi-square for other parameters, using the Statistical Package for Social Sciences (SPSS) Chic Ill. version 11. Relative risk (RR) and 95% confidence interval (CI) were calculated using the method described by Newcombe-Wilson.^10^

## Results

The age distribution in the two groups (pregnant and non-pregnant controls) was similar, with most women in the age group 20–35 years (91.3 vs 85.7%, respectively), as shown in Table 1. However, most of the patients in the control group were nulliparous (48/70, 68.6%), while parity in the pregnant group ranged between one and three offspring (41/69, 59.4%) (Table 1). There were 33, 28 and eight pregnant women in the expected gestational age groups of < 28 weeks, 28–36 weeks and > 36 weeks, respectively. Table 2 shows the summary of clinical and ECG parameters in the two groups with the relative risk, confidence intervals and *p*-values.

**Table 1.** Age And Parity Group Distribution

		*Pregnant (n = 69)*	*Non-pregnant (n = 70)*
Age (years)	< 20	1	2
	20–35	63	60
	> 35	5	8
Parity	0	23	48
	1–3	41	16
	> 3	5	6

**Table 2.** Summary Of Clinical And ECG Findings

	*Pregnant (n = 69)*	*Non-pregnant (n = 70)*	*RR (95% CI)*	p-*value*
Mean pulse rate	84 ± 11.05	75.27 ± 8.51	–8.73 (–12.04– –5.42)	0.043
DBP < 60 mmHg	64.7% (45)	24.3% (17)	2.685 (1.716–4.204)	< 0.005
SBP (90–120 mmHg)	80.9% (56)	78.6% (55)	1.033 (0.874–1.221)	0.704
Cardiac findings	41.2% (29)	12.9% (9)	3.156 (1.610–6.189)	< 0.0005
Mean ECG heart rate	88.34 ± 11.46	75.16 ± 12.22	–13.18 (–17.15– –9.21)	0.0215
Sinus tachycardia	8.7% (6)	2.9% (2)	3.044 (0.636–14.562)	0.266
Sinus bradycardia	0	5.7% (4)	–	0.132
PR < 0.12 s	0	1.4% (1)	–	–
PR > 0.20 s	1.5% (1)	2.9% (2)	0.507 (0.047–5.466)	0.999
QRS > 0.12 s	0	1.4%	–	–
Normal QRS axis	100%	100%	–	–
LVH (RI > 12 mm)	10.2% (7)	0	0.087 (0.019–0.155)	0.0189
All LVH criteria	18.8% (13)	7.1% (5)	2.638 (0.994–7.002)	0.0399
RVH	0	0	–	0
Rsr′ (mostly lead III)	5.8% (4)	14.3% (10)	0.406 (0.134–1.232)	0.0964
Rsr′ in avF	20.3% (14)	5.1% (4)	3.551 (1.230–10.252)	0.0105
ST segment – isoelectric line (J junction on isoelectric line)	97.1% (67)	75.7% (53)	1.283 (1.116–1.473)	< 0.0005
Mild ST elevation, (Negroid-pattern ST segment)	2.9% (2)	24.3% (17)	0.119 (0.029–0.497)	< 0.0005
T-wave inversion – lead III ± any other lead	23.2% (16)	10.0% (7)	2.319 (1.018–5.284)	0.0364
Tall and broad T waves in V_2_– V_6_	5.8% (4)	18.6% (13)	0.312 (0.107–0.910)	0.0215
APCS	4.3% (3)	2.9% (2)	1.522 (0.262–8.828)	0.987
VPCS	2.9% (2)	2.9% (2)	–	0.622
Path Q waves	0	0	–	–
APCS and VPCS together	7.3% (5)	5.8% (4)	1.268 (0.355–4.525)	0.982
Prolonged QTc	4.3% (3)	8.6% (6)	0.493, (–0.550–1.583)	0.505

Percentage of distribution (absolute number of patients). RR = relative risk, CI = confidence interval; DBP = diastolic blood pressure; SBP = systolic blood pressure; LVH = left ventricular hypertrophy; RVH = right ventricular hypertrophy; APCS = atrial premature contractions; VPCS = ventricular premature contractions.

Diastolic blood pressure (DBP) of less than 60 mmHg was found in 64.7% of the pregnant group, versus 24.3% of the control group (RR = 2.685, CI = 1.716–4.204, *p* < 0.005). When the patients were grouped according to age, statistical significance (for DBP < 60 mmHg) was reached for the age group 20–35 years (*p* < 0.005). Parity however did not affect DBP. Systolic blood pressure (SBP) was normal in both groups, ranging between 90 and 120 mmHg without any significant difference between the two groups.

On physical examination, findings ranging from grades 1–3 tricuspid systolic murmurs to loud P2 sounds were found in 41.2% of the pregnant group, whereas only grade 1 apical systolic murmur and occasional missed beats were found in 12.9% of the control group (RR = 3.156, CI = 1.610–6.189, *p* = 0.0005). Expected gestational age did not affect clinical findings (*p* = 0.738), even when EGA grouping based on trimester was used (*p* = 0.391) (Table 3).

**Table 3.** Distribution Of Significant Factors In Pregnant Group According To Trimester Of Pregnancy

	*3rd trimester*		*2nd trimester*		*1st trimester*		p*-value (Yates’ chi-square)*
	*Normal*	*Abnormal*	*Normal*	*Abnormal*	*Normal*	*Abnormal*	
Mean pulse rate	7	1	26	2	31	2	0.942 (0.119)
Mean ECG heart rate	7	1	25	3	31	2	0.945 (0.114)
Diastolic blood pressure	4	4	19	9	21	12	0.866 (0.287)
Cardiac findings	4	4	18	10	17	16	0.752 (0.571)
LVH (RI > 12 mm)	8	0	25	3	29	4	0.911 (0.186)
All LVH criteria	8	0	22	6	28	5	0.651 (0.86)
Rsr′ in aVF	3	0	19	9	28	5	0.417 (1.75)
ST-segment isoelectric line	8	0	28	0	28	5	0.196 (3.259)
Mild ST elevation (Negroid pattern)	8	0	26	2	33	0	0.564 (1.15)
T-wave inversion – lead III	8	0	21	7	24	9	0.494 (1.41)
Tall and broad T waves in V_2_–V_6_	8	0	26	2	32	1	0.932 (0.14)

All patients in both groups were in sinus rhythm. Sinus arrhythmia was found only in two non-pregnant patients. Mean ECG heart rate in the pregnant and control groups were 88.34 ± 11.46 and 75.16 ± 12.22 bpm, respectively. Sinus tachycardia was rare in both groups (8.7% in pregnant vs 2.9% in controls). However, the increase in ECG heart rate in the pregnant group compared with the controls was significant (RR = –13.18, CI = –17.15 to –9.21, *p* = 0.020). The mean ECG heart rate and pulse rate were higher among the pregnant subjects than controls (88.34 ± 11.46; 84.03 ± 11.05 vs 75.16 ± 12.22; 75.27 ± 8.51 bpm, *p* < 0.05 respectively).

The frontal-plane QRS axis was normal in all pregnant patients and non-pregnant controls. LVH using Sokolow-Lyon criteria revealed no significant difference in prevalence between the two groups; however using Araoye’s criterion in blacks (RI > 12 mm), the pregnant subjects had a higher prevalence of LVH than the normal controls (0.087, CI = 0.019–0.155, *p* = 0.0189). The LVH regressed in one of the two patients who reported for follow up eight weeks post partum; the other five patients were lost to follow up.

Non-specific intraventricular conduction defect was found in 5.8% of the pregnant group in the form of Rsr′, mostly in lead III, against 14.3% in the control group. Similarly Rsr′ was found in lead avF in 20.3% of the pregnant group, against 5.1% of the control (RR = 3.551, CI = 1.230–10.252, *p* = 0.0105).

Isolated atrial and ventricular ectopics were found in 7.3% of the pregnant group and 5.8% of the controls. First-degree atrioventricular block (PR > 0.20 s) was rare in both the pregnant and control groups (1.5 vs 2.9%, respectively). No other form of arrhythmia was found in either group.

Mild ST-segment elevation (J junction of the ST segment arising from within 1 mm of the isoelectric line, otherwise known as one of the ‘normal variants’ or the ‘normal Negroid pattern’ in blacks11) was found in 2.9% of the pregnant patients, against 24.3% in the control group (RR = 0.119, CI = 0.029–0.497, *p* < 0.0005). Isoelectric ST segment was also commoner in the pregnant subjects than the controls (97.1 vs 75.7%, RR = 1.283, CI = 1.116–0.473, *p* < 0.0005). Incidence was however less in the patients in the parity group above three (60%) when compared with 100% occurrence in the other parity groups: parity 0 and parity one to three (*p* < 0.005) (Table 4).

**Table 4.** Distribution Of Significant Factors In Pregnant Group According To Parity Status

	0 parous		1–3 parous		> 3 parous		*p*-value (Yates’ chi-square)
	Normal	Abnormal	Normal	Abnormal	Normal	Abnormal	
Mean pulse rate	21	2	39	2	4	1	0.925 (0.155)
Mean ECG heart rate	21	2	38	3	3	2	0.317 (2.292)
Diastolic blood pressure	16	7	27	14	3	2	0.984 (0.033)
Cardiac findings	13	10	22	19	3	2	0.971 (0.058)
LVH (RI > 12 mm)	21	2	36	5	5	0	0.978 (0.044)
All LVH criteria	20	3	33	8	5	0	0.856 (0.31)
Rsr′ in aVF	18	5	32	9	5	0	0.843 (0.34)
ST-segment isoelectric line	23	0	41	0	3	2	< 0.005 (13.50)
Mild ST elevation (Negroid pattern)	23	0	39	2	5	0	0.599 (1.023)
T-wave inversion – lead III	18	5	30	11	5	0	0.730 (0.63)
Tall and broad T waves in V_2_–V_6_	21	2	39	2	5	0	0.909 (0.191)

T-wave inversion in lead III ± any other lead was commoner in the pregnant group than the controls (23.2 vs 10%, RR = 2.319, CI = 1.018–5.284, *p* = 0.0364). By contrast, tall and broad T waves in V_2_–V_6_ occurred more commonly in the control group than the pregnant group (18.6 vs 5.8%, RR = 0.312, CI = 0.107–0.910, *p* = 0.0215).

The QTc was prolonged in a minor proportion of both the pregnant and control groups (4.3 vs 8.6%). The prolongation was however in the range of 0.46–0.47 s.

## Discussion

This study showed that pregnancy in Nigerian women might be associated with cardiac and electrocardiographic changes, including low DBP, systolic ejection murmur, higher heart rate, normal frontal-plane QRS axis, rarity of Negroid-pattern ST elevation and significant LVH based on Araoye’s criterion among blacks.

The low DBP was expected, because pregnancy reduces systemic vascular resistance and afterload, as a result of peripheral vasodilatation and the low resistance, high flow circulation of the uterus and placenta.^3^ In this study, significantly lower DBP was nearly three times more common in the pregnant patients than in the controls, and this was supported by other studies.^2^,^3^ Furthermore, by eight weeks’ gestation, the systemic vascular resistance fell by 70% of its preconception value.^12^

The common clinical findings in this study were tricuspid systolic murmur ranging from grade 1–3 and loud P_2_. The significant cardiac findings were still observed in the pregnant group, even after controlling for the possible effect of the estimated gestational age. These clinical findings were observed because the majority of the pregnancy-induced changes, such as reduced systemic vascular resistance, increased cardiac output, increased stroke volume and reduced arterial pressure occur during the first eight weeks of gestation.^12^

The ECG heart rate reached statistical significance between the two groups. Pregnancy has been well known to cause an increase in heart rate but not to the level of tachycardia. Only 8.7% of the pregnant patients had sinus tachycardia, against 2.9% of the control group. This was supported by previous studies, which reported that pregnancy only marginally increased heart rate by about 10–20 beats/min.^1^-^3^

The frontal-plane QRS axis was normal in all pregnant subjects, as previously reported.^13^ Axis deviation was not found in any of the study participants. Other studies have reported left and right axis deviation associated with normal pregnancy.^3^,^5^,^13^ We suggest that population-specific differences may account for this variation.

The incidence of atrial and ventricular premature complexes during pregnancy is unknown.^13^ The low incidence in this study compared with what was obtained in normal non-pregnant subjects, as it is not unusual to find these occasional ectopics in normal non-pregnant subjects.^9^

The rarity of the ‘normal Negroid-pattern’ ST elevation in the study subjects might mean that the expected pregnancy-associated ST segment sagging, as previously reported in some studies,^5^ depressed the ST segment to the isoelectric line. T-wave inversion in lead III ± any other lead was about twice as common in the pregnant group as in the controls. This has been reported in previous studies and is attributable to outward and upward shift of the cardiac apex by the enlarging uterus.^5^

In this study, no case of atrial fibrillation or flutter, other supraventricular tachyarrhythmias (SVT) or ventricular tachycardia was found. These conditions are rare in normal pregnancies and their presence should raise the suspicion of underlying severe cardiac disease during pregnancy.^13^

Non-specific intraventricular conduction defect (in avF) was found more frequently in the pregnant group (3.551, CI = 1.230–10.252, *p* = 0.0105). Similarly, tall and broad T waves in V_2_–V_6_ were found less commonly in the pregnant group than in the control group (0.312, 0.107–0.910, *p* = 0.0215). These had not been previously reported. We suggest the possibility of population-specific, pregnancy-related ECG changes. Further studies are needed to clarify this.

LVH determined from Araoye’s criterion was higher in prevalence among the pregnant subjects, based on identification with increased voltage in the R wave of lead 1 > 12 mm (0.087, CI = 0.019–0.155, *p* < 0.05). This was in support of previous studies, which had demonstrated that the heart is enlarged by both chamber dilatation and hypertrophy as a result of the haemodynamic changes that occur in pregnancy.^5^ Acute physiological LVH can occur rapidly during a normal human pregnancy, as an adaptive response to increased preload and cardiac work.^14^ This can be demonstrated during the second trimester and is most marked at the end of pregnancy.^15^ Even in a first pregnancy, the cardiovascular adaptation (LVH inclusive) begins early, can persist postpartum and appears to be enhanced by a subsequent pregnancy.^16^

The follow up of the patients eight weeks postpartum with a repeat ECG revealed normal voltage in the ECG in one out of two subjects who reported back for follow up. Reversal of chamber and hypertrophic changes of normal pregnancy has been shown to occur from a variable period of eight weeks to more than one year post delivery, due to the reversal of the haemodynamic changes associated with pregnancy.^14^-^18^ However, the proportion of subjects who reported back was too small to draw a meaningful conclusion on this and further studies are therefore suggested.

In Table 4, multiparity (> three) showed statistical significance in only the ST-segment isoelectric line parameter (*p* < 0.005), where three out of five in the more-than-three parity group were affected. The volume-overloaded state (with increased preload) of pregnancy causes physiological LVH;^17^ after the first pregnancy, subsequent pregnancies have been shown to enhance this.^16^ Similarly, as mentioned, the expected pregnancy-associated ST-segment sagging depressed the ‘Negroid pattern’ ST-segment elevation to the isoelectric line. LVH is responsible for the ST segment sagging, hence the parity-related enhancement of the physiological LVH would account for more multiparous women having their ST segment on the isoelectric line.

However, findings from our study were not in conformity with above arguments, as fewer patients in grand multiparous groups had demonstrable ST isoelectric lines. Echocardiographic indices could elucidate more correctly these haemodynamic changes and clarify this grey area.

The major limitation in this study was the small sample size and this is evident in the wide range demonstrated in the various confidence intervals.

## Conclusion

This study has provided data on the common cardiovascular and ECG findings in healthy pregnant women in Nigeria. The most common findings on physical examination were low diastolic blood pressure and systolic ejection murmur. There were also some distinctive ECG features, which may help to differentiate cardiac disease in pregnancy from normal cardiac findings in our practice area. In the pregnant Nigerian woman, normal frontalplane QRS axis, normal PR interval and ST segment arising from the isoelectric line are more or less the rule. LVH based on Araoye RI > 12 mm could be seen in a few others, while all forms of arrhythmia were rare.
